# Response of cyanobacterial mats to ambient phosphate fluctuations: phosphorus cycling, polyphosphate accumulation and stoichiometric flexibility

**DOI:** 10.1038/s43705-023-00215-x

**Published:** 2023-01-25

**Authors:** Laura Jentzsch, Hans-Peter Grossart, Sascha Plewe, Dirk Schulze-Makuch, Tobias Goldhammer

**Affiliations:** 1 Department of Ecohydrology and Biogeochemistry, Leibniz Institute of Freshwater Ecology and Inland Fisheries, 12587 Berlin, Germany; 2 Astrobiology Research Group, Zentrum für Astronomie und Astrophysik, Technische Universität Berlin, 10623 Berlin, Germany; 3 Department of Plankton and Microbial Ecology, Leibniz Institute of Freshwater Ecology and Inland Fisheries, 16775 Stechlin, Germany; 4 Institute of Biochemistry and Biology, Potsdam University, 14476 Potsdam, Germany; 5 Department of Marine Geology, Leibniz Institute for Baltic Sea Research Warnemünde, 18119 Rostock, Germany; 6 German Research Centre for Geosciences (GFZ), Section Geomicrobiology, 14473 Potsdam, Germany

**Keywords:** Microbial ecology, Biogeochemistry, Biofilms, Microbial ecology, Microbial ecology

## Abstract

Cyanobacterial mats inhabit a variety of aquatic habitats, including the most extreme environments on Earth. They can thrive in a wide range of phosphorus (P) levels and are thus important players for ecosystem primary production and P cycling at the sediment-water interface. Polyphosphate (polyP), the major microbial P storage molecule, is assigned a critical role in compensating for phosphate fluctuations in planktonic cyanobacteria, but little is known about potentially analogous mechanisms of mat-forming cyanobacteria. To investigate acclimation strategies of cyanobacterial mats to fluctuating phosphate concentrations, laboratory batch experiments were conducted, in which the cosmopolitan mat-forming, marine cyanobacterium *Sodalinema stali* was exposed to low dissolved P concentrations, followed by a P pulse. Our results show that the cyanobacteria dynamically adjusted cellular P content to ambient phosphate concentrations and that they had accumulated polyP during periods of high phosphate availability, which was subsequently recycled to sustain growth during phosphate scarcity. However, following the depletion of dispensable cellular P sources, including polyP, we observed a reallocation of P contained in DNA into polyP, accompanied by increasing alkaline phosphatase activity. This suggests a change of the metabolic focus from growth towards maintenance and the attempt to acquire organic P, which would be naturally contained in the sediment. P overplus uptake following a simulated P pulse further suggests that *Sodalinema*-dominated mats exhibit elaborated mechanisms to cope with severe P fluctuations to overcome unfavourable environmental conditions, and potentially modulate critical P fluxes in the aquatic cycle.

## Introduction

Cyanobacterial mats are benthic, microbial assemblages of photoautotrophic, chemoautotrophic and heterotrophic microorganisms with cyanobacteria as the main mat-builder that commonly thrive at the sediment-water interface (SWI) [1, 2]. They are embedded in a matrix of extracellular polymeric substances (EPS), creating a protected microenvironment with chemical conditions that can greatly differ from the overlying water body [3]. Cyanobacterial mats developed early in life history, representing among the oldest sedimentological fossils on Earth [4, 5], and persist until today. They occur in a variety of habitats in marine and freshwater ecosystems [6], including extreme environments such as hypersaline lakes [7] and hot springs [8]. Unique geochemical changes in the geologic record have been linked to the intricate advent of cyanobacteria, like the “Great Oxidation Event” in the Paleoproterozoic [9] and even today, they are still important for ecosystem productivity. Many strains are capable of fixing atmospheric nitrogen and thrive also under nutrient-poor conditions, whereby particularly cyanobacterial mats significantly contribute to primary production [10], building an important C source at the base of many aquatic food webs. However, their capability to synthesize toxins makes them key players in harmful algae blooms that pose a threat to particularly nutrient-rich, but also oligotrophic water bodies worldwide [11].

Phosphorus (P) is an essential element for life and is considered the ultimate limiting nutrient for aquatic primary producers [12]. Since most aquatic habitats are characterized by temporally varying phosphate concentrations, cyanobacteria have developed different biochemical strategies to cope with such fluctuations and allow growth even at low phosphate availability. These include an increase in P uptake rate and affinity [13], the expression of enzymes like Alkaline Phosphatase (APase) for mobilizing dissolved organic P [14], the substitution of phospholipids with sulphur- and nitrogen-containing membrane lipids [15], and the synthesis and recycling of polyphosphate (polyP) within the cells [16, 17]. PolyP is a major P and energy reserve in microorganisms, consisting of few to hundreds phosphate monomers linked by phosphoanhydride bonds that are often stored in association with cations in cellular granules [18]. The biochemical mechanism of polyP-based energy transfer is postulated to be very similar to the ADP/ATP system and involves hydrolytic cleavage of polyP chains via polyphosphate kinases [19] and is thus considered a plausible precursor for macromolecules in prebiotic times [20]. Besides being important for buffering nutrient P fluctuations and regulating the energy metabolism of microorganisms, polyP plays additional roles in cellular functions, including stress response (oxidative-, osmotic- and heat stress), stationary-phase adaptations, metal chelation, biofilm development and quorum sensing, as well as enhancing cellular adaptations to extreme environments [18, 21, 22].

The current literature describes three biochemical mechanisms of polyP synthesis—luxury uptake, overplus uptake, and deficiency response. Under excess phosphate conditions, polyP is accumulated over the current metabolic demand via “luxury uptake” and stored as polyP to be utilized when P becomes scarce [16, 23, 24]. When phosphate is resupplied to P-starved cells, they rapidly accumulate polyP at higher levels than obtained during luxury uptake, termed “overplus uptake” [25]. A third mechanism, known as “P deficiency response”, was more recently shown to also accumulate polyP at low P conditions [23]. While this typically coincides with increased APase activity, sulfolipid production and reduced RNA synthesis, the reasons causing polyP accumulation under severe P stress remain unresolved [26, 27, 23, 28, 29]. The relative accumulation of such biosynthetic P-rich molecules leads to dynamic cellular C:N:P ratios and opposes the traditional assumption of constant stoichiometry in oceanic plankton, known as the Redfield Ratio [30]. Variations from this ratio (106 C:16 N:1 P) are considered indicative of nutrient limitation [17] but also affected by the current growth phase of the cells, induced by allocation changes in the biosynthetic machinery, e.g., towards P-rich rRNA for protein synthesis at high growth rates [31].

Benthic microorganisms are particularly important for the P balance of many aquatic ecosystems, as they directly control P biogeochemistry at the SWI. PolyP contained in settling seston or benthic microorganisms can be released at the SWI upon cell death and lysis, acting as a P source for the benthic food chain [32]. On the other side, elevated phosphate concentrations in combination with Ca^2+^ contained in pore water can trigger apatite precipitation that ultimately removes P from the biosphere, and thus polyP is assigned a critical role in the P cycle of both, marine and freshwater systems [33]. Furthermore, sediment particles containing inorganic or organic P phases stick to the EPS and are trapped within the cyanobacterial mat. The diurnal metabolic cycle of respiration and photosynthesis yields alternating redox and pH conditions, which favours P release from such phases and provides an additional P source in otherwise oligotrophic systems, leading to substantially enriched phosphate concentrations within well-developed mats [3].

Like the more frequently studied planktonic cyanobacteria, benthic cyanobacterial mats display key communities in the aquatic P cycle. In contrast to planktonic cyanobacteria, they closely interact with the sediment that can act both, as a sink and source for P. In-situ polyP accumulation and recycling by cyanobacterial mats can thus be expected to critically impact P cycling at the SWI. This further leads to the question whether these fundamental mechanisms of P uptake, storage, and turnover of planktonic cyanobacteria can also be found in their benthic counterparts. This requires clarification for a more comprehensive understanding of global P dynamics in aquatic environments.

It has been recently demonstrated that polyP plays a vital role in other benthic systems as stream periphyton, suggesting it to be of likewise relevance also to cyanobacterial mats [28]. However, there are currently no studies investigating the fundamental intracellular P cycling processes in cyanobacterial mats during acclimation to P fluctuations. Hence, qualitative and quantitative investigations on polyP dynamics and stoichiometric flexibility under controlled laboratory conditions are needed to obtain insights into the benthic P cycle. Therefore, the response of a ubiquitous benthic mat-forming cyanobacterium, *Sodalinema stali*, to ambient P fluctuations was monitored in batch incubation experiments. It was tested whether the cells are capable of accumulating polyP, while addressing the following questions: [1] if they do so by luxury- or overplus uptake, or as a deficiency response [2], how they adapt their cellular stoichiometry, [3] whether proliferation can be sustained at low phosphate availability and [4] how they allocate available P resources.

## Methods

### Experimental setup and sampling

*Sodalinema stali*, formerl*y known as Microcoleus/Coleofascicculus chthonoplastes* [34], globally dominates cyanobacterial mats in marine, hypersaline, as well as terrestrial habitats [6] and can therefore be considered a model organism for cyanobacterial mats. The marine, non-axenic *Sodalinema stali* strain 31.92 [35] had been isolated from Mellum Island (North Sea) and was obtained from the Culture Collection of Algae of the Göttingen University (SAG), Germany. It is stated as “mutualized with non-specified bacterial or other type of contamination”, whereby *Sodalinema* dominated the biomass. The laminated texture that is typically observed in natural cyanobacterial mats due to the contribution of a diverse microbial community lacked during cultivation. The observed non-laminated mucilaginous structure formed by *Sodalinema* can be considered a precursor for a natural laminated cyanobacterial mat and is therefore referred to as a biofilm. The culture was inoculated with fresh BG11 + medium 5 days before the experiments. The biofilm was homogenized by shaking and washed before incubation as triplicates in a total volume of 4 l in either standard (175 µmol/l P = high P control experiment) or P-depleted (3.36 µmol/l P = low P experiment) BG11 + growth medium. (Table S1). Low P concentration was chosen to match the one (3.0 µmol/l P) applied in a freshwater *Coleofascicculus chthonoplastes* laboratory experiment [36]. Aiming to monitor the cellular response to consistent P exhaustion (low P) and constant P excess (high P control), the chosen P concentrations appear high, albeit reasonable, keeping in mind that P concentrations in thick cyanobacterial mats could be much higher compared to the overlying water body (320-fold) [3]. Biofilms were grown at 22 °C for 12 weeks under continuous shaking and illumination using glass beads as a substrate. A phosphorus pulse after 8 weeks was simulated by exchanging the P-depleted medium of the low P experiment with standard BG11 + medium, while the standard BG11 of the high P control experiment was exchanged with P-free BG11 + medium. At selected times, biofilm fragments were collected carefully by pipetting and homogenized by shaking (fragmented suspension) for subsequent analyses of APase activity, polyP content, and C:N:P ratios. Further, the medium was sampled and analysed for pH, soluble reactive phosphorus (SRP), ammonium (NH_4_), and dissolved inorganic carbon (DIC). Biofilm proliferation was monitored photographically and classified as either “growth” or “no-growth” (Fig, S1).

### SRP, NH_4_ and DIC

SRP and NH_4_ content of filtered samples (0.2 µm cellulose acetate membrane filters pre-washed with water) was determined photometrically by segmented flow analysis (SEAL AutoAnalyzer 3 HR). High NO_3_ concentrations of >15 mmol/l prevented potential N-limitation. DIC was determined by thermal combustion infrared spectroscopy after acidification (Shimadzu TOC-L) in filtered samples (0.45 µm cellulose acetate membrane pre-washed with water). Analytical precision of the SPR, NH_4_, and DIC measurements was calculated from the standard deviation of repeat standard measurements along with the samples and was <3%.

### APase activity

APase activity was determined photometrically, following the method proposed by Adams 2008 [14]. Briefly, 0.5 ml of the fragmented suspension was added to 0.5 ml of a 400 mM Tris-HCL buffer (pH 8.5) containing 7.2 mM para-Nitrophenyl phosphate (p-NPP), followed by incubation at 25 °C for 15 min in an orbital thermo mixer. APase hydrolysed p-NPP to p-Nitrophenol during incubation, which was measured photometrically at 410 nm (Horiba Aqualog) in the supernatant after centrifugation at 12000 × *g*. APase activity was normalized to dry weight after washing the remaining cell pellet four times with ultrapure water and drying it overnight at 60 °C. APase activity was calculated as µmol p-NPP hydrolysed per hour per litre of water (µmol/h/L).

### Cellular C:N:P molar ratio

The fragmented suspension was washed four times in ice-cold ultrapure water by centrifugation at 2450 × *g* and dried overnight at 60 °C. For total P determination, dried samples were digested to SRP by K_2_S_2_O_8_ addition at 120 °C in an autoclave. SRP was subsequently determined photometrically at 880 nm, following the addition of molybdenum blue and ascorbic acid similar to Murphy and Riley 1962 [37]. Analytical precision was <3% as tested by parallel measurements of photometric calibration standards. Bulk total nitrogen and carbon were determined by thermal combustion at 1020 °C using an elemental analyser (Thermo Scientific Flash EA) calibrated against in-house reference materials L-tyrosine and urea. Analytical precision was determined by repeat measurements of L-tyrosine along with biofilm samples and found to be <3%.

### PolyP staining

PolyP granules were visualized under an epifluorescence microscope, following DAPI-staining (D9542) [38]. Biofilm fragments were fixed using a 4% formaldehyde solution and stored at room temperature for visualization on the following day. Briefly, samples were incubated in a 0.3% Triton-X-100 solution for 5 min to permeabilize the cells and subsequently stained with a DAPI (20 µg/ml)-Mcllvaine solution (pH 7) for 30 min in the dark. Under UV excitation, polyP exhibits a yellow-greenish fluorescence colour at a 526 nm emission maximum (Fig. S2a), while DNA exhibits a blue fluorescence colour at a 461 nm emission maximum [39]. In individual cases, however, additional false staining of yellow-fluorescing elongated cell’s pole membranes was observed (Fig. S2b).

### PolyP mapping

PolyP mapping is based on automated identification of polyP granules and manual acquisition of the total cell area in the examined image. Three RGB images (2592 × 2048 pixels, scale:200 pixels ~10 µm) per sample, each of individually adjusted illumination time, were used for automated polyP identification (Fig. S2a). Image processing using the Fiji software (Fiji ImageJ v1.53k) included an initial background subtraction (rolling ball radius: 20 pixels), followed by conversion of the RGB image to a L*a*b* colour space. The a* channel, highlighting dark polyP granules on a light background, was subjected to an automated Yen segmentation (Fig. S2b: pre-PolyP image). False selected cell’s pole membranes were excluded by subtracting elongated particles (Aspect Ratio = 2–Infinity, Min Feret diameter = 0–0.7), followed by a watershed transformation from the selection twice (Fig. S2c: polyP image). The occupied area by polyP in the resulting image was determined and normalized to the respective cell area of each image, which was manually mapped in Adobe Photoshop (CS4) and displayed in % (Fig. S2d). The accuracy of this method was tested by manually removing the residual false positive selections, which revealed the automated method to be <3% less accurate. This loss in accuracy was considered negligible.

### Scanning electron microscopy (SEM)

SEM analysis was performed on a Zeiss MERLIN VP compact at the Leibniz Institute for Baltic Sea Research Warnemünde (IOW). Selected samples were washed four times in ultrapure water, dried at 20 °C and coated with chrome on a polycarbonate filter (0.2 µm) prior to analysis. Elemental distribution was analysed via Energy Dispersive X-Ray Analyses (EDX) by conducting line scans along cyanobacterial filaments, using the software OXFORD Aztec 3.3. Measurements were performed under high vacuum conditions with an acceleration voltage of 15 kV.

## Results

**Fig. 1:**
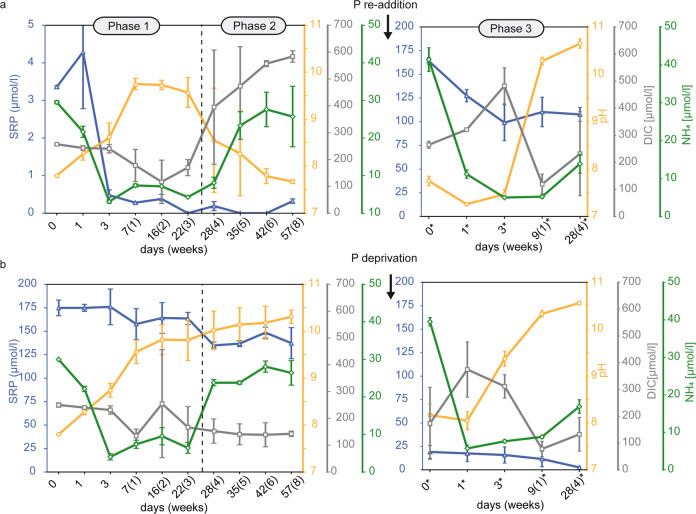
Medium composition at different biofilm development phases during the 12 weeks experiment. Numbers marked by a star symbol in Phase 3 indicate days after P re-addition or P-deprivation at day 57. **a** The low P experiment is characterized by proliferation in Phase 1 (3 weeks), pausing proliferation in Phase 2 (5 weeks) and proliferation after P re-addition in Phase 3 (4 weeks), while the high P control experiment (**b**) is characterized by proliferation in all Phases (1–3). Error bars display standard deviation of triplicates.

**Fig. 2:**
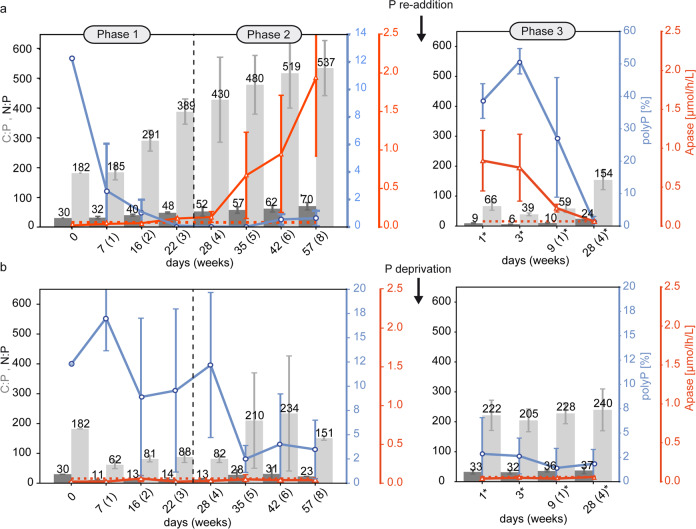
Cellular C:N:P ratio, polyphosphate (polyP) accumulation and alkaline phosphatase (APase) activity at different biofilm development phases during the 12 weeks experiment. Numbers marked by a star symbol in Phase 3 indicate days after (**a**) P re-addition in the low P experiment or (**b**) P-deprivation in the high P control experiment at day 57. The red dashed line indicates the threshold of elevated APase activity. Error bars display the standard deviation of triplicates.

**Fig. 3:**
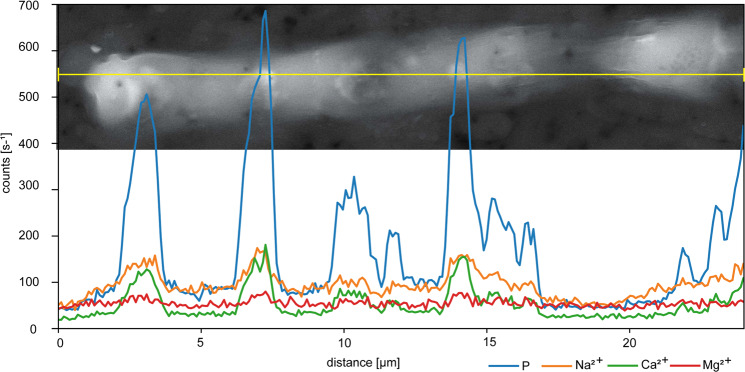
SEM/EDX analyses of a Sodalinema stali filament one day after P re-addition. Polyphosphate (polyP) granules are characterized by high P peaks, associated with peaks in Na^2+^, Ca^2+^ and Mg^2+^.

**Fig. 4:**
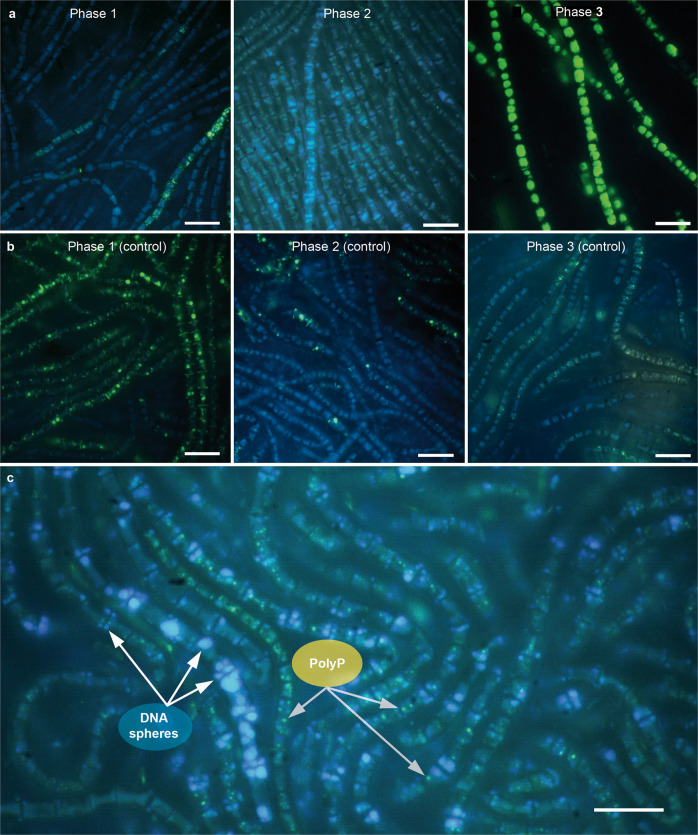
Examples of qualitative microscopic observations at different biofilm development phases. DAPI-polyphosphate (polyP) fluoresces in a yellow-green colour, DAPI-DNA fluoresces in a blue colour. **a** Phases of the low P experiment. Phase 1: Heterogeneous polyP accumulation in individual filaments, Phase 2: DNA accumulation into globular spheres and heterogeneous polyP accumulation at the end of Phase 2, Phase 3: Homogeneous polyP accumulation in all cells, being of noticeable size (**b**) Phases of the high P experiment. Phase 1: Homogeneous polyP accumulation in all cells, Phase 2–3: Heterogeneous polyP accumulation in individual cells, (**c**) Close up of Phase 2 of the low P experiment showing DNA spheres preferentially accumulating at the cell’s polar membranes and adjacent to polyP. White bar indicates a scale of 10 µm.

Acclimation of *Sodalinema stali* to ambient P concentrations was monitored over a 12-week period (85 days), including a simulated P pulse in the low P experiment and P deprivation in the high-P experiment after 8 weeks. Three distinct development phases were identified based on variations in (1) proliferation (Fig. S1) and medium composition (Fig. 1), (2) polyP accumulation, APase expression and cellular composition (Fig. 2), and (3) qualitative microscopic observations (Figs. 3, 4).

### Phase 1

Phase 1 lasted for three weeks from day 1–22 and was characterized by biofilm proliferation in both experiments, at low P (3.36 µmol/l) and high P (175 µmol/l) control concentrations. In the low P experiment, most phosphate had been consumed within the first three days, indicated by the decrease of SRP down to 0.48 µmol/l, while proliferation and photosynthesis were sustained during the entire Phase 1, indicated by photometric observations (Figure S 1) and increasing pH (Fig. 1a), respectively. Fluorescence microscopy revealed that simultaneously with SRP uptake, the initially contained polyP reserves of 12.3% were recycled to sustain growth (Fig. 2a). Despite the major depletion of SRP and polyP, the constant C:N:P ratio of ≈187:31:1 since incubation and the relatively low APase activity, being below the maximal values detected in the control experiments (0.06 µmol/L/h), do not indicate severe P stress in the first week of Phase 1 (Fig. 2a). However, the rapidly increasing C:N:P ratio in weeks 2 (291:40:1) and 3 (389:48:1) of Phase 1 (Fig. 2a) suggests the mobilization of other cellular P-containing compounds to maintain growth at the onset of P stress. In the control experiment under high P availability, no signs of P stress were observed, and proliferation was maintained under continuously high SRP levels and increasing pH (Fig. 1b). Further, cellular P content increased relative to day 0, indicated by narrow C:N:P ratios (62:11:1–88:14:1) during Phase 1, while polyP content remained at high levels (8.89–17.04%) (Fig. 2b).

### Phase 2

Phase 2 lasted for 5 weeks from day 23–57 and was characterized by pausing proliferation in the low P experiment (Fig. S1). Previous depletion of the SRP pool, polyP reserves and other cellular P-containing compounds prevented further proliferation. APase activity increased during Phase 2 to a final activity of 1.94 µmol/h/L (Fig. 2a), indicating increasingly severe P stress. Limited photosynthesis was indicated by a decrease of the pH to 7.67 (Fig. 1a) and the loss of photosynthetic pigments, which led to a change from green towards a yellow-green colour of the biofilm (Fig. S1). Increasing DIC (584 µmol/l) and NH_4_ (26.1 µmol/l) concentrations until the end of Phase 2 (Fig. 1a) suggested decaying organic matter and mineralization, respectively. The slowly but progressively increasing C:N:P ratio throughout weeks 4 (430:52:1), 5 (480:57:1), 6 (519:62:1) and 8 (537:70:1) of Phase 2 (Fig. 2a) suggests the mobilization of less easily dispensable cellular P-containing compounds compared to Phase 1, to support fundamental cellular processes others than growth under severe P stress. Despite progressive P stress at the end of Phase 2, polyP accumulation of 0.55% and 0.64% at day 42 and 57, respectively, was observed (Fig. 2a). Although this increasing trend appears to be small, polyP content was significantly elevated compared to the previous weeks 4 and 5 when cells were completely devoid of polyP. Fluorescence microscopy revealed that the usually homogeneously distributed, blue-fluorescing DNA accumulated into globular spheres, which occurred throughout the cells but preferentially accumulated near the cell’s pole membranes. These DNA spheres first occurred on day 28 when the polyP pool had been exhausted (Fig. S3), but also adjacent to polyP at the end of Phase 2 (Fig. 4a: Phase 2, Fig. 4c). In the control experiment, no signs of P stress were observed in Phase 2, and proliferation was maintained under high SRP levels and a pH increase to 10.3 (Fig. 1b). However, higher C:N:P ratios (151:23:1–234:31:1) in correlation with lower polyP levels (2.50–4.02%) than in Phase 1 (62:11:1–88:14:1) were observed between day 35 and 57 (Fig. 2b).

### Phase 3

Phase 3 lasted for four weeks and was initiated at day 57 by P re-addition to the P–stressed cells of the low P experiment and by P–deprivation from the high P control experiment, leading to an SRP concentration of 164 µmol/l and 19.1 µmol/l, respectively (Fig. 1a, b). Phase 3 lasted from day 0*–28* (numbers marked by a star symbol in Phase 3 indicate days after P re-addition or P-deprivation at day 57) and was characterized by proliferation in both experiments (Fig. S1). Despite the high phosphate availability following P re-addition, elevated, albeit decreasing, APase activity until day 9* (Fig. 2a) indicated ongoing P stress. Within the first three days, SRP was consumed rapidly down to a concentration of 99.0 µmol/l, while the reduced pH (7.3–7.5) indicated that only limited photosynthesis occurred during this period (Fig. 1a). However, SRP uptake resulted in narrow C:N:P ratios at day 1 (66:9:1) and 3 (39:6:1), while polyP was accumulated simultaneously by 38.6% and 50.8%, respectively (Fig. 2a). In the remaining Phase 3, SRP levels remained constant (≈109 µmol/l) and indicated a period of low phosphate uptake, while the increasing pH up to 10.7 indicated high photosynthesis rates (Fig. 1a). Hence, to maintain subsequent proliferation until the end of Phase 3, the cellular P content was reduced, leading to a C:N:P ratio of 154:24:1, while polyP was recycled down to 1.37% (Fig. 2a). Following P-deprivation in the high P experiment, no signs of P stress were observed in Phase 3, and proliferation was maintained under progressive SRP consumption down to 2.59 µmol/l and increasing pH up to 10.6 (Fig. 1b). Both, polyP content (1.83–2.86%) and C:N:P ratio (205:32:1–240:37:1) remained unaffected by the reduced phosphate availability (Fig. 2b).

### Qualitative microscopic observations

In addition to DAPI staining, the presence of polyP was further confirmed by SEM/EDX analyses (Fig. 3), which revealed the association of the cations Na^2+^, Ca^2+^ and Mg^2+^ with P, which is characteristic for cellular polyP inclusions [18].

Microscopic analyses further revealed that polyP was not always distributed equally between the cells, except when polyP content and cellular P content were highest at the beginning of Phase 3 of the low P experiment (Fig. 4a), where polyP were of noticeable size, and in Phase 1 of the high P experiment (Fig. 4b). A heterogeneous polyP distribution of individual filaments filled with polyP next to polyP-free filaments was observed in Phase 1 of the low P experiment (Fig. 4a). Further, only individual cells of filaments next to polyP–free cells, which led to high standard deviations, were observed in Phase 2 and at the end of Phase 3 of the low P experiment (Fig. 4a, c) and in Phase 2 and 3 of the high P experiment (Fig. 4b).

## Discussion

Our findings highlight the critical role of polyP in *Sodalinema stali*-formed cyanobacterial mats, as it was dynamically accumulated and recycled during acclimation to P fluctuations.

### Cellular response to progressive P starvation

**Fig. 5:**
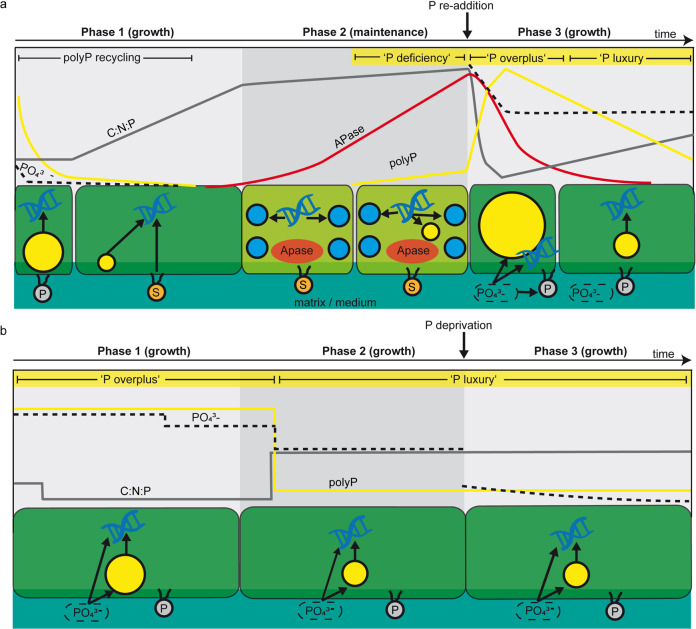
Schematic interpretation of cellular phosphorus (P) cycling in a cyanobacterial mat, based on significant changes of the monitored parameters (arbitrary units). **a** At low P availability, initially contained polyphosphate (polyP) was recycled simultaneously with phosphate uptake to sustain growth at constant C:N:P ratios. Further proliferation at the onset of P stress in Phase 1 was sustained by mobilization of cellular P, e.g. phospholipids, which led to rapidly increasing C:N:P ratios. Severe P stress in Phase 2, indicated by increasing APase activity, prevented proliferation and photosynthesis, indicated by a loss of green chlorophyll pigments. PolyP accumulation by deficiency response occurs with severely increasing P stress, whereby globular DNA accumulation indicates the allocation of P contained in DNA into polyP. P re-addition to the P-stressed cells in Phase 3 triggered overplus uptake and narrow C:N:P ratios, transitioning to luxury uptake at higher C:N:P ratios following polyP recycling. **b** At high P availability, polyP in Phase 1 was accumulated by overplus uptake at narrow C:N:P ratios, transitioning to luxury uptake at higher C:N:P ratios during polyP recycling in Phase 2. P-deprivation in Phase 3 did not affect the cells, which we attributed to a sufficient amount of phosphate in the residual medium or within the biofilm matrix. Arrows indicate phosphorus transformation processes, whereby arrows pointing towards DNA represent cell growth. Yellow granules = polyP, blue granules = globular DNA spheres, P = phospholipids, S = substitute lipids.

Analogous to planktonic cyanobacteria, growth under low P availability could be sustained by recycling polyP, which acted as a primary P source (Fig. 2a) [16, 23, 24]. We further attribute the rapid reduction of easily dispensable cellular P-containing compounds to the substitution of cellular phospholipids with S- or N-containing membrane lipids to maintain growth at the onset of P stress (Fig. 2a) [15, 23]. However, the exhaustion of this easily dispensable P pool limited proliferation in Phase 2, and the metabolic strategy switched from a focus on growth towards maintenance (Fig. 5). The interpretation of prevailing cellular processes based on our results is graphically summarized and explained in detail below (Fig. 5).

Severe P stress in Phase 2 was indicated by the colour change from green towards yellow-green (Fig. S1) and increasing APase activity (Fig. 2a). The colour change suggested the loss of photosynthetic pigments [40], but we could not clarify whether this occurred through active cellular pigment reduction or degradation of available chlorophyll e.g., by oxidation. The increasing APase activity (Fig. 2a) suggested that *Sodalinema stali* is capable of hydrolysing organic P [14]. Even though APase expression did not trigger proliferation, it likely hydrolysed a potentially available organic P pool, as increasing DIC, NH_4_ and decreasing pH indicated progressive decay and remineralisation of organic matter (Fig. 1a). This suggests that in analogous oligotrophic environments with often fluctuating conditions, the strategy has to be maximizing the utilization of external P sources contained in organic and inorganic sediment particles that get trapped in the EPS [41]. The sediment can contain large amounts of organic P [42] and the fluctuating physico-chemical gradients in the EPS matrix due to high daytime pH and low oxygen conditions at night, facilitate P desorption from metal oxides, leading to higher dissolved phosphate concentrations within the mat, compared to the overlying water body [3]. However, alternating redox conditions at the SWI could also trigger polyP release from benthic microorganisms to the sediments, where it could act as a P source for the benthic food-chain, or ultimately trigger the formation of mineral P phases [32], to sustainably remove P from the aquatic cycle. Either way, we suggest that polyP-containing cyanobacterial mats critically impact P fluxes at the SWI.

With persisting severe P stress and increasing APase activity in Phase 2, polyP accumulation as a deficiency response was observed (Fig. 2a), which has been reported from planktonic cyanobacteria of different habitats [24, 29, 23], as well as stream periphyton [28]. However, the reasons causing this deficiency response remain unresolved. In marine phytoplankton of the oligotrophic Sargasso Sea, Martin et al. [23] excluded that polyP-rich cells were in a perpetual overplus state with ‘undetectable’ pulses driving this state and suggested that polyP accumulation occurred as a cellular stress response. In other studies, reduced biosynthesis of P-rich rRNA coincided with deficiency responses [26, 28] and led to the suggestion that polyP accumulation at P concentrations below a certain threshold required for growth occurs because of P allocation changes away from growth and towards storage. Further, APase can hydrolytically cleave phosphate groups from nucleic acids and convert DNA-lipid-P to DNA-lipids, which were shown to self-assemble into globular lipid-based DNA micelles [43]. These preferentially anchor on cell membranes [44], and indeed, such DNA spheres were found to accumulate at the cell’s polar membranes in our experiments adjacent to polyP during deficiency response (Fig. 4a: Phase 2,c). Therefore, we suggest that intracellular P recovery by cleavage from P-rich DNA and reallocation to polyP, and potentially reduced rRNA synthesis [31], is also a strategy in benthic mats of *Sodalinema stali* as a response to severe P stress when P availability is too low to sustain growth. This supports the theory of a reallocation of resources away from growth towards flexibly available P and energy storage. Such direct intracellular P cycling could be beneficial to help retain P within the cyanobacterial population; while external P moieties such as dissolved organic P within the matrix can act as an additional P source, they are also likely to be subject to nutrient competition between cyanobacteria and other organisms inhabiting the matrix.

Such effects of potential interactions in terms of nutrient competition or provision between cyanobacteria and mutualistic microorganisms contained within the same EPS matrix are difficult to assess and we cannot exclude some potential effects on our results. However, mutualistic microorganisms that are naturally contained in many cyanobacterial or algal cultures are often critical for metacommunity functioning and hence, working with axenic mat-forming strains may even further falsify any obtained results. Furthermore, microscopic analyses revealed that *Sodalinema* always dominated the biomass and hence, it is here considered reasonable to work with a non-axenic culture.

### Cellular response to a simulated P pulse

In P-deficient cells, the affinity of the P uptake system is typically increased to maximize P uptake for future pulses [13, 45]. The simulated P pulse to the P-stressed cells in Phase 3 led to a rapid increase of the cellular P content by 1260% relative to C within 3 days (Fig. 2a), whereby P was accumulated to a significant part as polyP, which is characteristic for overplus uptake [25]. Many different types of oligotrophic aquatic habitats experience only temporal P pulses, e.g., from redox changes at the benthic interface leading to P release from the sediment [32], storm run-off [28], upwelling [46], or excretions of aquatic animals [47]. The capability of microorganisms to immediately take up, store, and efficiently re-use this P by overplus uptake is hence of critical importance for a population to sustain a potential subsequent period of low P availability. Overplus uptake is typically accompanied by the overall slow growth of the population and cellular recovery from P starvation, including ultrastructural organization and recovery of the photosynthetic apparatus [48]. This took one week after re-feeding of P-starved *Nostoc* sp. PCC 7118 cells [48]—a timeframe very similar to the delayed onset of photosynthesis observed in our study, indicated by the elevated pH at day 9 (Fig. 1a). Regarding overplus-triggering mechanisms following P pulses, Solovochenko et al. [48] suggested that overplus uptake occurs due to a delayed down-regulation of high-affinity P_i_ transporters, which are active during P starvation, and emphasized the simultaneous advantage of osmotically inert polyP accumulation as a response to dramatically high phosphate concentrations in the cells. Even though APase levels declined following our experimental P re-addition, they were significantly elevated for at least 9 days (Fig. 2a). As our experimental design involved replacing the medium with APase-free, BG11 + medium after Phase 2, we assume that the APase detected in Phase 3 was actively produced, and we conclude that previously relevant, low-P response mechanisms are slowly disengaged with some sort of lag, even when ambient P is repleted. Following cellular recovery, *Sodalinema* now recycled stored polyP instead of further accumulating it during the transition from overplus-to luxury uptake, which was reflected in the increasing C:N:P molar ratios and decreasing polyP levels without significant additional phosphate uptake (Figs. 1a, 2a, 5).

### Qualitative observations on polyP distribution

Most methods applied to analyse polyP in microorganisms are quantitative and do not contain information on its spatial distribution within a population. The here observed variable distribution of polyP between the cells during luxury uptake and deficiency response, as well as the retention of polyP in few individual filaments during polyP recycling in Phase 1 of the low P experiment (Fig. 4) suggests strategies of either slow growth with a retention of polyP, or of high growth with polyP recycling. This was also suggested for cells of a unicellular *Synechocystis sp*. PCC 6803 population during overplus uptake [47]. In contrast, polyP in our experiment was distributed homogeneously between all cyanobacterial cells during overplus uptake (Fig. 4a: Phase 3, Fig. 4b: Phase 1). Yet, we are unaware of any polyP distribution study in multicellular or mat-forming cyanobacteria and hence, further mechanisms of interactions, e.g., cell-to-cell communication [49, 50], might also contribute to purposeful differentiation of cells or filaments within a common matrix.

In summary, our study shows that the mat-forming *Sodalinema stali* (1) is capable of luxury uptake, overplus uptake and deficiency response with a heterogenous polyP distribution during polyP recycling, luxury uptake and deficiency response, while (2) dynamically adjusting cellular P content to changing phosphate concentrations. (3) Proliferation is sustained under the expense of polyP, followed by P acquisition from other easily dispensable cellular P-containing compounds under the onset of P stress. (4) Further, biosynthetic allocation changes away from growth towards maintenance with relative polyP accumulation at the expense of P-rich DNA are conducted under severe P stress. Our findings demonstrate the extraordinary capabilities of mat-forming cyanobacteria to adapt their P acquisition strategies to strong P fluctuations. While lasting proliferation under P limitation requires the mobilization of additional P sources through regeneration of P from particulate matter, the transition to net P accumulation under excess ambient P is rapid and effective. Since current projections of climate and land use change include intensified pulses of P load to aquatic ecosystems [50], e.g., through external input from surplus of agriculture fertilizer, inefficient wastewater treatment plants, and internal loads via the mobilization of legacy P, these P ‘bioaccumulators’ could form an important component in P remediation by temporarily accumulating P within the mat, and synthesizing polyP that could ultimately stimulate the formation of mineral P phases to sustainably remove P from the aquatic cycle.

## Supplementary information

Supplementary Information

## Data Availability

The datasets generated during and/or analysed during the current study are available in the FRED repository, https://fred.igb-berlin.de/data/package/805.
