# Supplementary material for: Response of cyanobacterial mats to ambient phosphate fluctuations: phosphorus cycling, polyphosphate accumulation and stoichiometric flexibility
Source: ISME Commun. 2023 Jan 25;3:6. doi: 10.1038/s43705-023-00215-x (PMC9876960; doi:10.1038/s43705-023-00215-x)
Supplement: Supplementary file 1 — Supplementary Information [file 43705_2023_215_MOESM1_ESM.docx]

# Supplementary Information

The following supplementary information belong to the article:

Response of cyanobacterial mats to ambient phosphate fluctuations: phosphorus cycling, polyphosphate accumulation and stoichiometric flexibility

Laura Jentzsch^1,2^, Hans-Peter Grossart^3,4^, Sascha Plewe^5^, Dirk Schulze-Makuch^2,3,6^, Tobias Goldhammer^1^

1. Department of Ecohydrology and Biogeochemistry, Leibniz Institute of Freshwater Ecology and Inland Fisheries, 12587, Berlin, Germany
2. Astrobiology Research Group, Zentrum für Astronomie und Astrophysik, Technische Universität Berlin, 10623 Berlin, Germany
3. Department of Plankton and Microbial Ecology, Leibniz Institute of Freshwater Ecology and Inland Fisheries, 16775, Stechlin, Germany
4. Institute of Biochemistry and Biology, Potsdam University, 14476, Potsdam, Germany
5. Department of Marine Geology, Leibniz Institute for Baltic Sea Research Warnemünde, 18119, Rostock, Germany
6. German Research Centre for Geosciences (GFZ), Section Geomicrobiology, 14473 Potsdam, Germany

Corresponding author: Laura Jentzsch, Email: [jentzsch@igb-berlin.de](mailto:laura.jentzsch@igb-berlin.de)

Table S 1: Composition of standard and modified (red colour) BG11+ growth medium

| **components** | **mg/L** |
| --- | --- |
| K_2_HPO_4_*3H_2_O | 40 / 0.68 |
| KCL | 0 / 25.7 |
| Na_2_CO_3_ | 20 |
| Fe-NH_4_-citrate | 6 |
| NaNO_3_ | 1500 |
| Mg(SO_4_)*7H_2_O | 75 |
| CaCl_2_*2H_2_O | 36 |
| Na_3_-citrate*2H_2_O | 6 |
| Na_2_-EDTA*2H_2_O | 0.1 |
| Vitamine B12 | 0.02 |
| H_3_BO_3_ | 2.9 |
| MnCl_2_*4H_2_O | 1.81 |
| ZnSO_4_*7H_2_O | 0.22 |
| Na_2_MoO_4_*2H_2_O | 0.39 |
| CuSO_4_*5H_2_O | 0.8 |
| Co(NO_3_)_2_*6H_2_O | 0.5 |
| NaCl | 10000 |


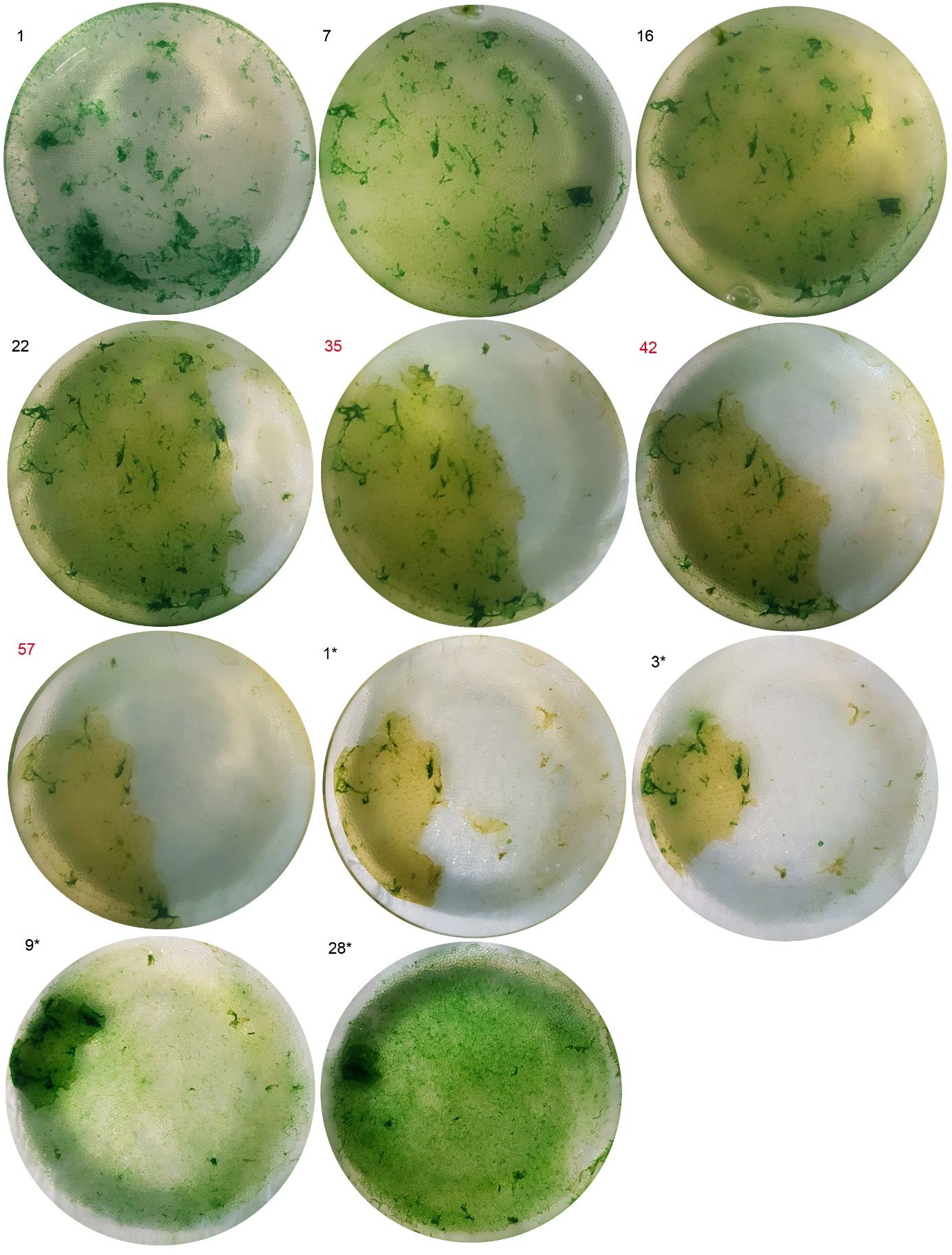


Figure S 1: Example of photometric monitoring of cyanobacterial mat proliferation at low ambient phosphate availability. Proliferation was observed between day 1–22 in Phase 1 and day 1–28 in Phase 3 (black day numbers), whereas proliferation paused in Phase 2 between day 35–57 (red day numbers)


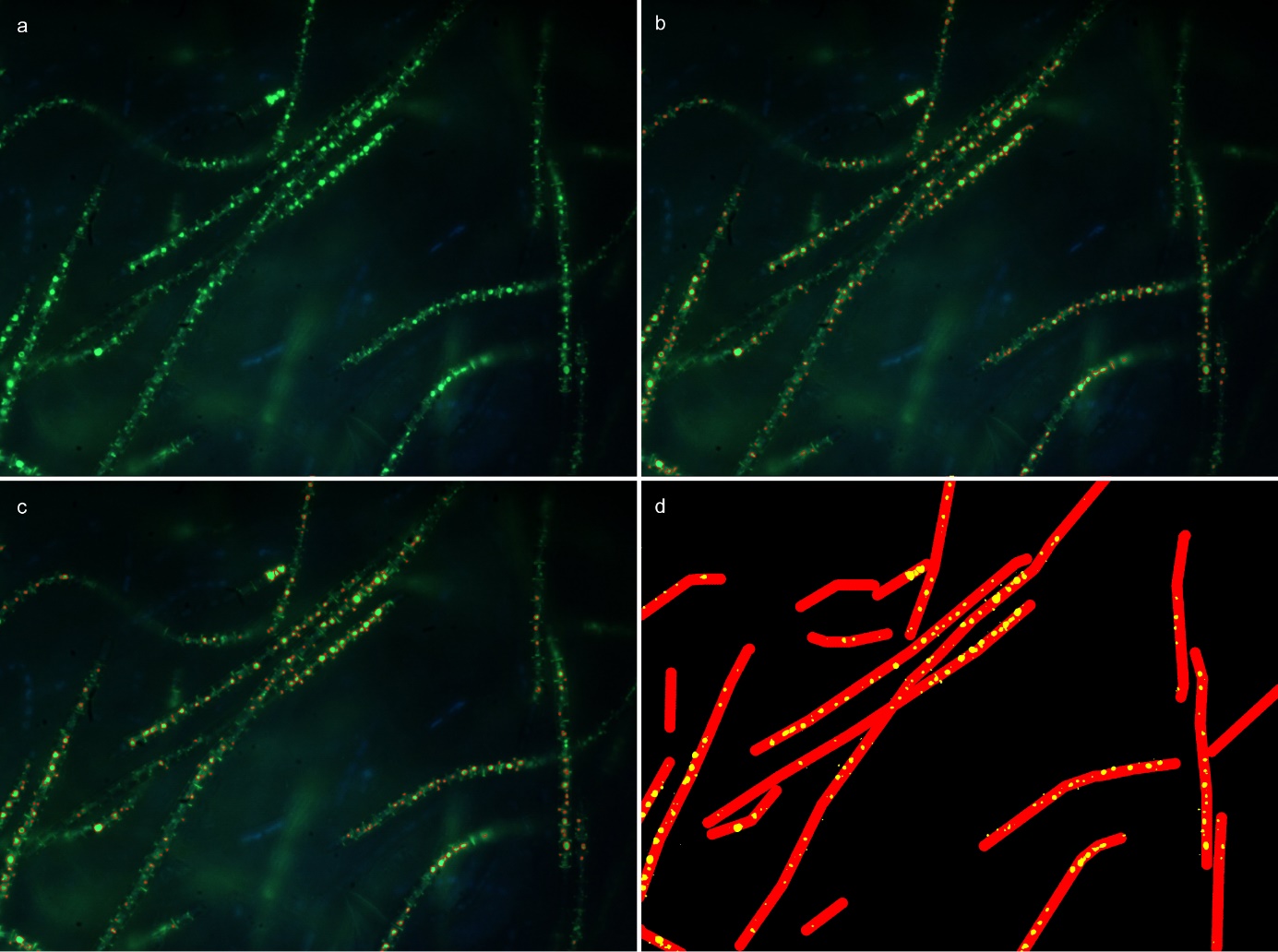


Figure S 2: Polyphosphate (polyP) Mapping. (a) Original fluorescence image (b) pre-polyP Image (c) polyP image after removed false-stained cell’s pole membranes (d) area occupied by polyP (yellow) and area occupied by cells (red)
